# The Correlation Between White Matter Hyperintensity Burden and Regional Brain Volumetry in Patients With Alzheimer's Disease

**DOI:** 10.3389/fnhum.2022.760360

**Published:** 2022-06-14

**Authors:** Zhiyu Cao, Yingren Mai, Wenli Fang, Ming Lei, Yishan Luo, Lei Zhao, Wang Liao, Qun Yu, Jiaxin Xu, Yuting Ruan, Songhua Xiao, Vincent C. T. Mok, Lin Shi, Jun Liu, The Alzheimer's Disease Neuroimaging Initiative

**Affiliations:** ^1^Department of Neurology, Sun Yat-sen Memorial Hospital, Sun Yat-sen University, Guangzhou, China; ^2^BrainNow Research Institute, Shenzhen, China; ^3^Department of Neurology, The Second Affiliated Hospital of Guangzhou Medical University, Guangzhou, China; ^4^Division of Neurology, Department of Medicine and Therapeutics, Gerald Choa Neuroscience Centre, Lui Che Woo Institute of Innovative Medicine, The Chinese University of Hong Kong, Hong Kong, Hong Kong SAR, China; ^5^Department of Imaging and Interventional Radiology, The Chinese University of Hong Kong, Hong Kong, Hong Kong SAR, China

**Keywords:** Alzheimer's disease, magnetic resonance imaging, brain atrophy, white matter hyperintensities, cholinergic pathway

## Abstract

**Background:**

White matter hyperintensities (WMHs) and regional brain lobe atrophy coexist in the brain of patients with Alzheimer's disease (AD), but the association between them in patients with AD still lacks comprehensive investigation and solid imaging data support.

**Objective:**

We explored whether WMHs can promote the pathological process of AD by aggravating atrophy in specific brain regions and tried to explain the regional specificity of these relationships.

**Methods:**

A sample of 240 adults including 180 normal controls (NCs) and 80 cases with AD were drawn from the ADNI database. T1-weighted magnetic resonance imaging (MRI) and T2-weighted fluid-attenuated MRI of the participants were downloaded and were analyzed using AccuBrain^®^ to generate the quantitative ratio of WMHs (WMHr, WMH volumes corrected by intracranial volume) and regional brain atrophy. We also divided WMHr into periventricular WMHr (PVWMHr) and deep WMHr (DWMHr) for the purpose of this study. The Cholinergic Pathways Hyperintensities Scale (CHIPS) scores were conducted by two evaluators. Independent *t*-test, Mann–Whitney *U* test, or χ^2^ test were used to compare the demographic characteristics, and Spearman correlation coefficient values were used to determine the association between WMHs and different regions of brain atrophy.

**Results:**

Positive association between WMHr and quantitative medial temporal lobe atrophy (QMTA) (*r*_*s*_ = 0.281, *p* = 0.011), temporal lobe atrophy (*r*_*s*_ = 0.285, *p* = 0.011), and insular atrophy (*r*_*s*_ = 0.406, *p* < 0.001) was found in the AD group before Bonferroni correction. PVWMHr contributed to these correlations. By separately analyzing the relationship between PVWMHr and brain atrophy, we found that there were still positive correlations after correction in QMTA (*r*_*s*_ = 0.325, *p* = 0.003), temporal lobe atrophy (*r*_*s*_ = 0.298, *p* = 0.007), and insular atrophy (*r*_*s*_ = 0.429, *p* < 0.001) in AD group.

**Conclusion:**

WMH severity tends to be associated with regional brain atrophy in patients with AD, especially with medial temporal lobe, temporal lobe, and insular lobe atrophy. PVWMHs were devoted to these correlations.

## Introduction

Alzheimer's disease (AD) is an age-related neurodegenerative disease with complex pathologies. Its main gross structural change is brain atrophy observed in MRI. Although aging can lead to a certain degree of brain atrophy, there are still significant differences between age-related brain atrophy and AD-related brain atrophy. Studies showed that age-related brain atrophy mainly appeared in temporal, frontal, and occipital lobes, while AD-specific brain atrophy was seen most impressively in the hippocampus, amygdala, and entorhinal cortex (Raji et al., 2009; Habes et al., 2016). Therefore, evaluating brain atrophy might be an effective method to estimate the appearance and progression of AD (Matsuda, 2016). Besides brain atrophy, cerebrovascular changes could also exist in the brain (Staffaroni et al., 2017). The intervention of cerebrovascular-related risk factors such as obesity, hypertension, diabetes, and lack of homocysteine could effectively reduce the incidence of AD and slow the cognitive decline in patients with AD (Hamel et al., 2016; Livingston et al., 2017). This indicated that there could be associations between AD and cerebral small-vessel disease, and cerebrovascular changes might be an initiating factor in the early stage of AD (Kester et al., 2014; Javanshiri et al., 2018).

White matter hyperintensities (WMHs) were identified as intensive signals on T2-weighted MRI sequences, especially in T2 fluid-attenuated inversion recovery (FLAIR). It was commonly considered to be a feature of aging and cerebrovascular changes (Wardlaw et al., 2013; Franchetti et al., 2020). Cohort studies suggested that WMHs were associated with different types of cognitive declines including vascular dementia and AD (Gordon et al., 2015; Bos et al., 2018; Georgakis et al., 2019; Hu et al., 2020; Wang et al., 2020a). Many cerebrovascular risk factors could contribute to the increased load of WMHs, and these factors might also increase the incidence of AD. A growing number of researchers hypothesized there might be a close relationship between AD and cerebral small vessel disease, with evidence from positron emission tomography (PET) that cerebral vascular changes have synergistic effects with Aβ and tau pathology (Haight et al., 2013; Provenzano et al., 2013). WMHs could also appear in the context of gliosis, axonal loss, and demyelination and are probably correlated with a more proliferative immune reactive environment, and these could also result in a neuronal loss (Schmidt et al., 2011).

White matter hyperintensities were associated with cognitive decline and the severity of AD and could predict the conversion from MCI to AD (Prasad et al., 2011). Some studies suggested that WMHs were related to the biomarkers of AD (Soldan et al., 2020). There might be a link between WMHs and AD, but the role of WMHs in the pathology of AD was unclear. Although many researchers wanted to ensure the relationship between WMHs and brain volumes, there was still no consensus. Some studies hypothesized that WMH volumes were positively correlated with gray matter reduction or hippocampal volume reduction, while others suggested that this association was not significant (Wen et al., 2006; Appel et al., 2009; Jang et al., 2013; Kandiah et al., 2015; Fiford et al., 2017; Vipin et al., 2018).

In this study, we used AccuBrain version 1.2^®^ to perform quantitative measures for brain structure, an FDA-approved software generated by BrainNow Medical Technology Limited, China. In the previous study, we found that AccuBrain^®^ could accurately segment and measure hippocampus volume like FreeSurfer, a well-used tool for brain structure measurement worldwide (Abrigo et al., 2019). We also proved that AccuBrain^®^ had good accuracy and reproducibility in segmenting and measuring WMHs, especially in 3D T2-weighted fluid-attenuated inversion recovery (T2-FLAIR) MRI (Guo et al., 2019; Wang et al., 2020b). Different from previous studies, this cross-sectional study not only explored the relationship between WMHs and quantitative medial temporal lobe atrophy (QMTA) but also analyzed the correlation between WMHs and atrophy of occipital, temporal, frontal, parietal, and insular lobe. The aim of this study was to explore whether WMHs were associated with the brain atrophic changes in AD and, thus, to guide the treatment of AD with the cerebrovascular lesion.

## Materials and Methods

### Subjects

A cohort of 240 adults aged from 57 to 91 years was acquired from the Alzheimer's Disease Neuroimaging Initiative (ADNI, http://adni.loni.usc.edu/) database, including 160 normal controls and 80 cases with AD matched with age, sex, and education. Led by Principal Investigator Michael W. Weiner, MD, the ADNI was launched in 2003, and its aim was to decide whether serial MRI, PET, other biological markers, and clinical and neuropsychological assessment can be combined to measure the progression of mild cognitive impairment (MCI) and early AD. More details about ADNI can be found on the ADNI homepage.

### MRI Acquisition

Only subjects with T2-FLAIR images were selected for this cross-sectional study. Imaging was performed using 3T GE, Philips, or Siemens scanners with standardized scanning parameters. The T1-weighted (T1-W) images were acquired by sagittal volumetric magnetization prepared rapid gradient echo (MP-RAGE) or inversion recovery-fast spoiled gradient recalled (IR-SPGR) sequences. For T2-FLAIR images, some of them were conducted by sagittal 3D sequences and others by axial 2D sequences. More information on this MR protocol could be found on the ADNI website (http://adni.loni.usc.edu/methods/mri-tool/mri-analysis/).

### Quantitative Volumetry

In this study, all MR images were automatically segmented and quantified by AccuBrain version 1.2^®^ (an FDA-approved software generated by BrainNow Medical Technology Limited, China) (Abrigo et al., 2019). The following indexes reported using this cloud-based neuroimage analysis tool were recorded and statistically analyzed.

As the most characteristic structural change of AD, QMTA was calculated according to the ratio of inferior lateral ventricular volumes to hippocampus volumes.

The degree of atrophy of each brain lobe was calculated as the ratio of the volumes of cerebrospinal fluid (CSF) to brain parenchyma within the respective lobe. We generated temporal lobe atrophy, frontal lobe atrophy, parietal lobe atrophy, cingulate lobe atrophy, occipital lobe atrophy, and insular lobe atrophy in this study (Zhao et al., 2019).

**Figure 1:**
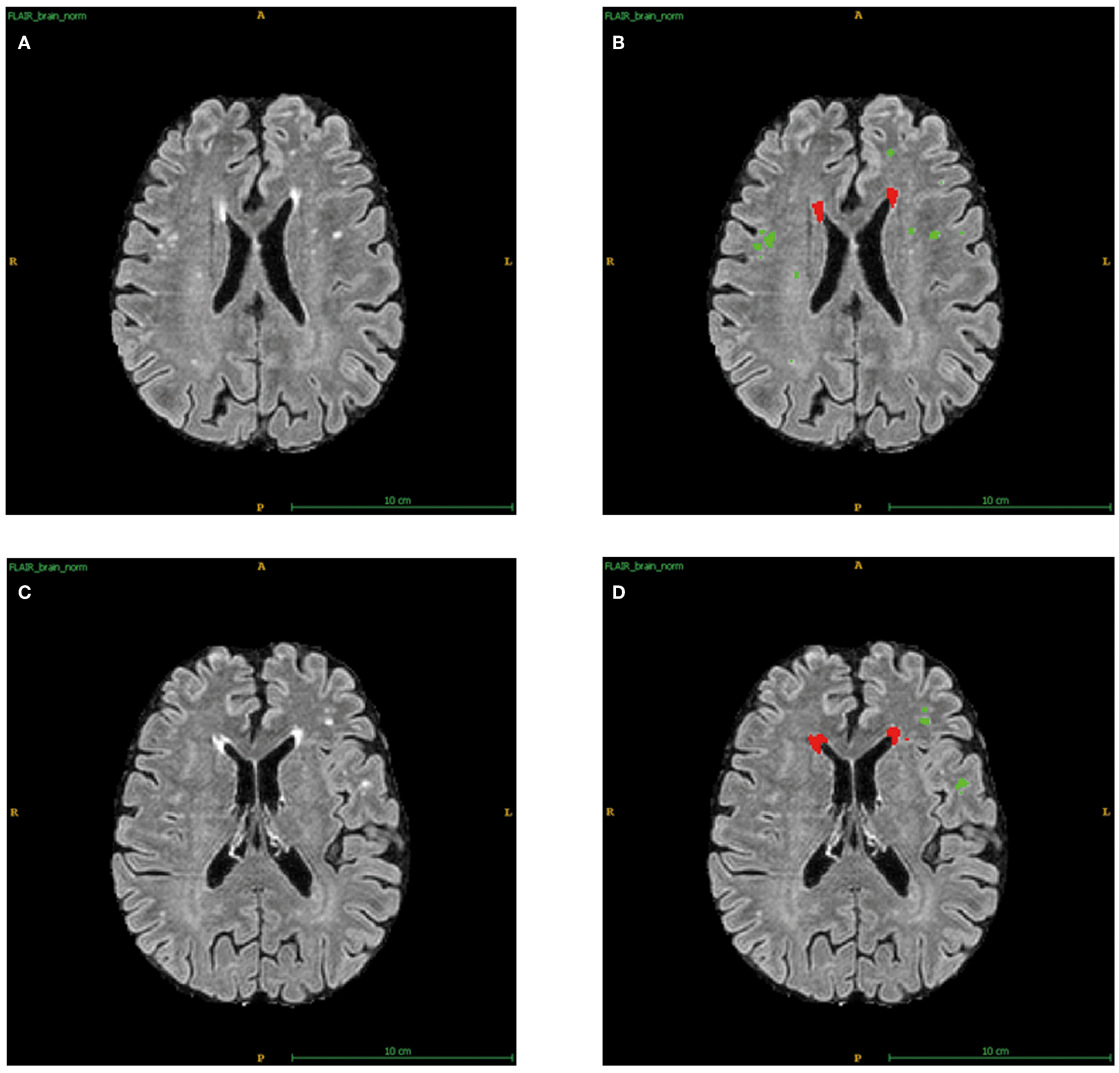
Automatic segments of PVWMHs and DWMHs applied by AccuBrain^®^. **(A)** The original T2-FLAIR images of sample 1. **(B)** Automatic division of PVWMHs (Red) and DWMHs (Green) for **(A)**. **(C)** The original T2-FLAIR images of sample 2. **(D)** Automatic division of PVWMHs (Red) and DWMHs (Green) for **(C)**.

As in previous studies, an automated WMH segmentation algorithm was used to quantify total WMH volumes for each subject (Shi et al., 2013). The measurement of WMHs using AccuBrain^®^ has been described in detail in previous studies (Guo et al., 2019). In short, segmentation was first performed on T1-W images, then T1-W and T2-FLAIR images were registered together, and WMHs were segmented from coarse to fine by mathematical morphology. Besides, like the Fazekas scales (Fazekas et al., 1987), periventricular WMHs (PVWMHs) and deep WMHs (DWMHs) were quantified separately using AccuBrain^®^ (Figure 1). Apart from the absolute WMH volumes, we calculated the WMH ratio using the intracranial volume (ICV) to reduce the influence of head size, which was called WMHr in this study.

### Visual Ratings

**Figure 2:**
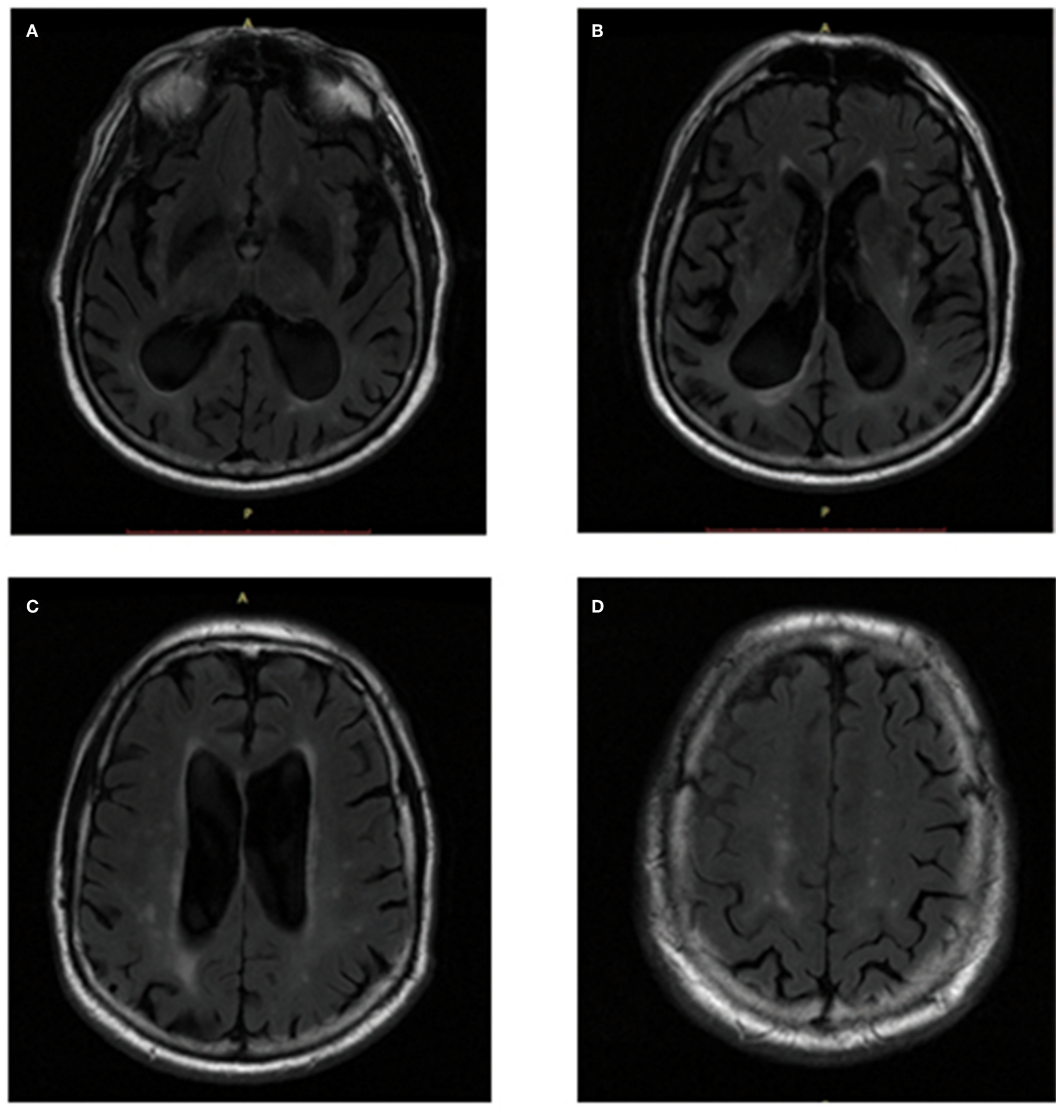
CHIPS scores showed in T2-FLAIR images. **(A)** Low External Capsule: Anterior (Right = 0, Left = 1, Factor = 4, Total = 4); Posterior (Right = 1, Left = 1, Factor = 4, Total = 8). **(B)** High External Capsule: EC Anterior (Right = 1, Left = 1, Factor = 3, Total = 6); EC Posterior (Right = 1, Left = 1, Factor = 3, Total = 6); Cingulate (Right = 0, Left = 0, Factor = 4, Total = 0). **(C)** Corona Radiata: CR Anterior (Right = 1, Left = 1, Factor = 2, Total = 4); CR Posterior (Right = 1, Left = 1, Factor = 2, Total = 4); Cingulate (Right = 0, Left = 0, Factor = 1, Total = 0). **(D)** Centrum Semiovale: Anterior (Right = 0, Left = 1, Factor = 1, Total = 1); Posterior (Right = 1, Left = 1, Factor = 1, Total = 2).

From the T2-FLAIR images, the Fazekas score was used to preliminarily evaluate the location and extent of WMHs of all subjects. PVWMHs were rated as 0: absence; 1: “caps” or pencil-thin lining; 2: smooth “halo” (6–10 mm regular margins); 3: irregular halo >10 mm or extending into the deep. DWMHs were rated as 0: absence; 1: multiple focal lesions>5; 2: beginning confluent lesions; 3: large confluent lesions (Fazekas et al., 1987, 1993). The Cholinergic Pathways Hyperintensities Scale (CHIPS) score (Bocti et al., 2005) was used to assess the WMH load in the cholinergic tracts (Figure 2). Based on the histochemistry tracing of cholinergic pathways in cerebral hemispheres, four index axial slices named low external capsule, high external capsule, corona radiata, and centrum semiovale were divided into ten regions and each region contributed by one factor. Each slice adopted a three-point system (0 = normal; 1 < 50.00% involvement; 2 ≥ 50.00% involvement), and the total score equaled the sum of the score of each region multiplied by the corresponding factor, for a total of 100 points (Selden et al., 1998). All visual ratings were performed by two trained neurologists, YRM (with 6 years of experience for visual ratings) and ZYC (with 3 years of experience for visual ratings).

### Statistical Analysis

Statistical analyses were performed using SPSS version 25.0 and R version 4.1.0. As age and years of education met normal distribution, an independent *t*-test was used to compare the demographic characteristics of NC and AD subjects. For other continuous variables that did not satisfy normal distribution, Mann–Whitney *U* test was used. Besides, we used χ^2^ test to decide the difference in sex, hypertension, hyperlipidemia, and diabetes composition in different groups. Interclass correlation coefficient (ICC) was used to evaluate the consistency between the two raters in CHIPS. Since the variables we used to reflect brain atrophy did not fit the normal distribution, we chose the Spearman correlation coefficient to determine whether there were associations between WMHs and regional brain atrophy. To balance the data distribution, we have log10-transformed our WMHr, PVWMHr, and DWMHr. The statistical description of the transformed data is listed in Supplementary Table 2. We used logarithmic values to draw scatter plots in the Supplementary Materials to roughly show these correlations. In the Spearman correlation analysis, we showed the original *p-*values. As we performed 7 pairwise comparisons per group, Bonferroni correction was used, and a *p-*value less than 0.0071 (0.05/7) was regarded as statistical significance after correlation. As Bonferroni was a conservative method, the correction might increase the false-negative rate. All statistical tests were two-tailed. Demographic comparisons were significant at *p* < 0.05.

## Results

### The Reliability of Visual Rating and Quantitative Volumetry

We used the ICC to evaluate the inter-rater repeatability between the two raters (Table 1). The ICCs between the two raters on the Fazekas scale were above 0.9 and above 0.85 in the CHIPS score, which meant that our visual scores were well repeatable. We also analyzed the correlation between quantitative WMHr and Fazekas scale separately in PVWMHs and DWMHs (Supplementary Table 1) and found that it was a moderate positive correlation between PVWMHs (*r*_*s*_ = 0.526, *p* < 0.001) and DWMHs (*r*_*s*_ = 0.474, *p* < 0.001).

**Table 1:** Inter-rater reliability of visual ratings.

**Scores**	**ICC**	***p* value**
**Fazekas scores**		
PVWMH	0.926	<0.001
DWMH	0.969	<0.001
**Slices of CHIPS score**		
Low external capsule	0.883	<0.001
High external capsule	0.933	<0.001
Corona radiate	0.883	<0.001
Centrum semiovale	0.948	<0.001
Total score	0.929	<0.001

CHIPS, the Cholinergic Pathways Hyperintensities Scale; ICC, interclass correlation coefficient. ^*^The p-values were obtained by the interclass correlation coefficient test.

### Demographic Information and Group-Wise Volumetric Difference

**Figure 3:**
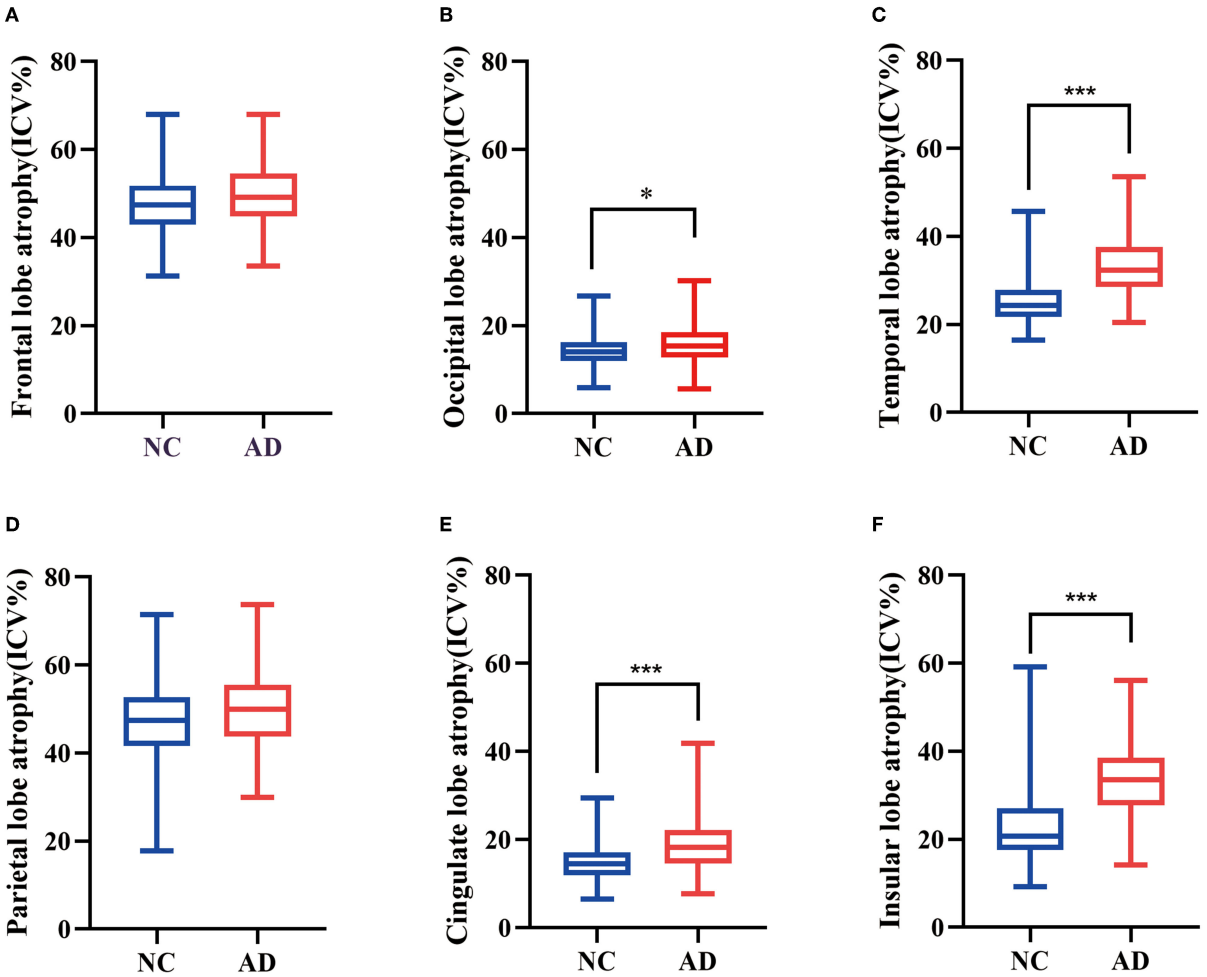
Box plots were used to show the difference of quantitative regional brain atrophy between AD and NC. Frontal lobe atrophy **(A)** and parietal lobe atrophy **(D)** showed no significant difference between the two groups. Occipital lobe atrophy **(B)**, temporal lobe atrophy **(C)**, cingulate lobe atrophy **(E)**, and insular lobe atrophy **(F)** were more serious in AD than NC. ^*^P < 0.05, ^***^P < 0.001.

A total of 240 participants including 160 NC subjects and 80 patients with AD were included in this study. Among these two groups, age, sex, and years of education were matched. As WMHs could be greatly influenced by vascular factors, we collected the history of diseases in the samples and found that there was no significant difference in the proportion of hypertension, diabetes, and hyperlipidemia between NC and AD groups. WMHr in the AD group were significantly higher than in the NC group, in which PVWMHs were found to be significantly increased in AD compared with NC *via* both visual ratings (*p* = 0.005) and quantitative measurement (*p* < 0.001) (Table 2). We used CHIPS scores to evaluate the extent of impairment of cortical cholinergic projections (Figures 2A–D) and found that CHIPS scores in AD were also higher than in NC (*p* < 0.001) (Table 2). We found that atrophy of occipital lobe (*p* = 0.008), temporal lobe (*p* < 0.001), cingulate lobe (*p* < 0.001), and insular lobe (*p* < 0.001) was severer in AD than NC (Figure 3).

**Table 2:** Demographic statistics and imaging-based quantifiers of NC and AD cohorts.

		**NC**	**AD**	
**Variables**		**(*n =* 160)**	**(*n =* 80)**	***p* value**
Age	Mean (SD)	74.18 (5.45)	74.71 (7.75)	0.539
Years of education	Mean (SD)	15.75 (2.42)	15.54 (2.58)	0.531
Gender (Female)	*N* (%)	73 (45.63)	35 (43.75)	0.783
MMSE	Mean (SD)	28.86 (1.28)	22.93 (2.92)	**<0.001**
ADAS	Mean (SD)	13.37 (4.53)	31.70 (7.70)	**<0.001**
**History of diseases**				
Hypertension	*N* (%)	67 (41.88)	34 (42.50)	0.926
Diabetes	*N* (%)	16 (10.00)	7 (8.75)	0.756
Hyperlipidemia	*N* (%)	84 (52.50)	50 (62.50)	0.141
**Visual ratings**				
PVWMHs	Mean (SD)	1.20 (0.61)	1.43 (0.60)	**0.005**
DWMHs	Mean (SD)	0.78 (0.81)	0.92 (0.81)	0.175
CHIPS	Mean (SD)	25.44 (11.70)	31.23 (8.16)	**<0.001**
**Quantitative imaging tests**				
QMTA	Mean (SD)	0.41 (0.15)	0.77 (0.38)	**<0.001**
DWMHr	Mean (SD)	0.08 (0.09)	0.08 (0.07)	0.074
PVWMHr	Mean (SD)	0.29 (0.35)	0.57 (0.52)	**<0.001**
WMHr	Mean (SD)	0.37 (0.41)	0.65 (0.54)	**<0.001**

NC, normal cognitively; AD, Alzheimer's Disease; ICV, intracranial volume MMSE, mini-mental state examination; ADAS, Alzheimer's disease assessment scale; WMHs, white matter hyperintensities; DWMHs, deep WMHs; PVWMHs, periventricular WMHs; CHIPS, the Cholinergic Pathways Hyperintensities Scale; DWMHr, quantitative DWMH ratio; PVWMHr, quantitative PVWMH ratio; WMHr, quantitative WMH ratio; QMTA, quantitative medial temporal atrophy. ^*^The p-values were obtained using the independent t-test (age and years of education) or χ^2^ (gender, hypertension, diabetes, and hyperlipidemia) test or Mann–Whitney U test (other variables).

### Correlation Analysis Between WMHr and Regional Brain Atrophy

We first conducted Spearman correlation before statistical correction. In the NC group, QMTA (*r*_*s*_ = 0.288, *p* < 0.001), temporal lobe atrophy (*r*_*s*_ = 0.225, *p* = 0.004), cingulate lobe atrophy (*r*_*s*_ = 0.199, *p* = 0.012), and insular atrophy (*r*_*s*_ = 0.392, *p* < 0.001) showed significantly positive association with WMHr. In the AD group, QMTA (*r*_*s*_ = 0.281, *p* = 0.011), temporal lobe atrophy (*r*_*s*_ = 0.285, *p* = 0.011), and insular atrophy (*r*_*s*_ = 0.406, *p* < 0.001) showed positive correlation with WMHr (Table 3; Supplementary Figure 1). After Bonferroni correction, only insular atrophy showed a correlation with WMHr in the AD group while QMTA, temporal lobe atrophy, and insular atrophy still showed a correlation in the NC group. By exhaustively analyzing the correlation between PVWMHr, DWMHr, and brain atrophy, we found that the correlation between PVWMHr and brain atrophy was almost consistent with that between WMHr and brain atrophy. After correction, PVWMHr were still positively correlated with QMTA (*r*_*s*_ = 0.325, *p* = 0.003), temporal lobe atrophy (*r*_*s*_ = 0.298, *p* = 0.007), and insular atrophy (*r*_*s*_ = 0.429, *p* < 0.001) in patients with AD (Table 4; Supplementary Figure 1). These results suggested that WMHs probably showed a positive correlation with brain atrophy in the medial temporal lobe, temporal lobe, and insular lobe in both NC and AD, but these correlations seemed not so remarkable. To further explain these findings, we then focused on WMHs in the cholinergic pathway.

**Table 3:** Correlation analysis between regional brain atrophy and WMHr.

	**NC**		**AD**	
	***r_***S***_***	***p* value**	***r_***S***_***	***p* value**
QMTA	0.288	<0.001^*^	0.281	**0.011**
Frontal Lobe Atrophy	−0.029	0.712	−0.115	0.312
Occipital Lobe Atrophy	0.042	0.598	0.037	0.747
Temporal Lobe Atrophy	0.225	0.004^*^	0.285	**0.011**
Parietal Lobe Atrophy	−0.147	0.063	−0.204	0.070
Cingulate Lobe Atrophy	0.199	**0.012**	0.072	0.523
Insular Atrophy	0.392	<0.001^*^	0.406	<0.001^*^

NC, normal cognitively; AD, Alzheimer's disease; WMHr, quantitative WMH ratio; QMTA, quantitative medial temporal atrophy; r_s_, Spearman r. The p-value was obtained by Spearman correlation coefficient values. ^*^means statistical significance after Bonferroni correction.

**Table 4:** Correlation analysis between regional brain atrophy and different types of WMHr.

	**NC**				**AD**			
	**PVWMHr**		**DWMHr**		**PVWMHr**		**DWMHr**	
	***r_***S***_***	***p* value**	***r_***S***_***	***p* value**	***r_***S***_***	***p* value**	***r_***S***_***	***p* value**
QMTA	0.325	<0.001^*^	0.017	0.828	0.325	0.003^*^	0.003	0.978
Frontal lobe atrophy	0.015	0.855	−0.081	0.306	−0.087	0.441	−0.142	0.208
Occipital lobe atrophy	0.103	0.195	−0.124	0.118	0.039	0.733	0.025	0.827
Temporal lobe atrophy	0.298	<0.001^*^	−0.022	0.784	0.298	0.007^*^	0.166	0.141
Parietal lobe atrophy	−0.117	0.142	−0.180	**0.023**	−0.204	0.070	−0.134	0.237
Cingulate lobe atrophy	0.207	**0.009**	0.099	0.215	0.121	0.284	−0.150	0.183
Insular atrophy	0.437	<0.001^*^	0.140	0.078	0.429	<0.001^*^	0.159	0.160

NC, normal cognitively; AD, Alzheimer's disease; WMHr, quantitative WMHs ratio; DWMHr, quantitative deep WMH ratio; PVWMHr, quantitative periventricular WMH ratio; QMTA, quantitative medial temporal atrophy; r_s_, Spearman r. The p-value was obtained by Spearman correlation coefficient values. ^*^means statistical significance after Bonferroni correction.

### Correlation Between CHIPS and Regional Brain Atrophy

The ICC between two evaluators was all greater than 0.85 on four index axial slices and total scores (Table 1). After analyzing the correlation between CHIPS and brain atrophy in different groups before correlation, we found that CHIPS scores were positively correlated with QMTA (*r*_*s*_ = 0.231, *p* = 0.039), temporal lobe atrophy (*r*_*s*_ = 0.272, *p* = 0.014), cingulate lobe atrophy (*r*_*s*_ = 0.239, *p* = 0.033), and insular atrophy (*r*_*s*_ = 0.281, *p* = 0.012) in AD, while these relationships were absent in QMTA and cingulate lobe atrophy in NC. In the NC group, only temporal lobe atrophy (*r*_*s*_ = 0.190, *p* = 0.016) and insular atrophy (*r*_*s*_ = 0.181, *p* = 0.022) were positively associated with CHIPS (Table 5). Although these correlations seemed not significant after Bonferroni correction, it could prompt the tendency that WMHs in the cholinergic tracts might affect brain atrophy.

**Table 5:** Correlation analysis between regional brain atrophy and CHIPS in NC and AD groups.

	**NC**		**AD**	
	***r_***S***_***	***p* value**	***r_***S***_***	***p* value**
QMTA	0.125	0.116	0.231	**0.039**
Frontal lobe atrophy	0.060	0.451	−0.010	0.933
Occipital lobe atrophy	0.117	0.140	−0.011	0.923
Temporal lobe atrophy	0.190	**0.016**	0.272	**0.014**
Parietal lobe atrophy	0.009	0.913	−0.200	0.075
Cingulate lobe atrophy	0.071	0.372	0.239	**0.033**
Insular atrophy	0.181	**0.022**	0.281	**0.012**

*NC, normal cognitively; AD, Alzheimer's disease; CHIPS, the Cholinergic Pathways Hyperintensities Scale; QMTA, quantitative medial temporal atrophy; r_s_, Spearman r. The p-value was obtained by Spearman correlation coefficient values*.

## Discussion

Alzheimer's disease is one of the most prevalent age-related neurodegenerative diseases with cognitive decline and brain atrophy. Since the disease is irreversible, it is important to find treatments in the early stages of AD to prevent its progression. In recent years, an increasing number of studies have begun to focus on the effects of vascular damage on AD. It was widely accepted that cerebral small vessel disease (SVD) and aging were the main causes of white matter lesions. Clinical and animal studies demonstrated that white matter lesions might be the starting point of AD and began to emphasize the contribution of vascular disease to AD progression (Rosenberg et al., 2016; Quintana et al., 2021). WMHs have been reported to independently predict the emergence of AD at least 10 years before the onset of clinical symptoms, suggesting that vascular factors might be an important target for preclinical detection and intervention (Mortamais et al., 2014).

There was a significant correlation between WMHs and cognitive decline in both AD and SVD, where brain atrophy often appeared (Pantoni, 2010; van den Berg et al., 2018). The clinical manifestation of AD was polymorphic, and clinical symptoms were likely to be influenced by different regions of WMHs and brain atrophy (Schmidt et al., 2011). In patients with AD, WMHs were positively correlated with cognitive decline and behavioral and psychological symptoms of dementia (BPSD), and MTA was associated with the cognitive decline and behavioral and psychological symptoms while frontal and insular connections were associated with the emotional expression in AD (Kandiah et al., 2014; García-Alberca et al., 2019; Jones et al., 2019; Wang et al., 2020a). Higher WMHs were also related to higher AD biomarkers such as Aβ (Alosco et al., 2018). One Harvard Aging Brain Study implicated that the vascular risk factor might lie upstream of Aβ deposition (Rabin et al., 2018). These studies supported that WMHs might play a key role in the early stage of AD. As we age, the decrease in blood vessel elasticity and hemodynamics could lead to cerebral ischemia, which not only affected the nutrient supply of cortical neurons but also affected the clearance of Aβ, thus aggravating the development of AD pathology (Vasileyko et al., 2010). Aβ deposition in brain parenchyma was considered to be the central part of the pathophysiological process such as increased tau phosphorylation and immune system activation, which resulted in neuron loss and consequently brain atrophy (Scheltens et al., 2016). Cerebral amyloid angiopathy (CAA) is a disease where vascular and AD pathologies coexisted. Studies have found that CAA could lead to cognitive decline, which might be achieved by reducing the thickness of the cortex (Subotic et al., 2021). A lot of anti-Aβ drugs were proposed but failed in clinical trials, so it is urgent to find new treatments to prevent AD progression in the early stage (Long and Holtzman, 2019).

Our research found that medial temporal lobe, cingulate lobe, temporal lobe, occipital lobe, and insular lobe atrophy were severer in patients with AD than in NC, which was partly consistent with previous studies (Fan et al., 2008). We also found that WMHs were positively correlated with QMTA, temporal lobe, and insular atrophy in both NC and AD groups before correction, which meant that WMHs tended to play a role in brain atrophy with or without AD pathologies. Interestingly, in this study, the relationship between PVWMHs and brain atrophy was consistent with that of WMHs. DWMHs did not demonstrate any correlation to brain lobe atrophy, which might be because PVWMHs could damage the cholinergic pathways that start from the nucleus basalis of Meynert in previous studies. Due to this, PVWMHs might play a more important role in the cognitive decline in AD than DWMHs (Swartz et al., 2003).

Our findings confirmed a probable relationship between WMHs and AD. Then, we tried to explain why the association between WMHs and brain atrophy occurred in specific brain regions. The cholinergic system, starting from the nucleus basalis of Meynert in the central nervous system, plays an important role in advanced cognitive activities such as learning, memory, and attention, through the transmission of cholinergic neurotransmitters. It often exists in the thalamus, striatum, limbic system, and neocortex (Hampel et al., 2018). It has been found that cholinergic WMHs were associated with AD signature brain structural changes including the atrophy of hippocampal, medial orbitofrontal, frontal, parietal, and temporal regions as well as worse cognitive functions especially processing speed (McNeely et al., 2015; Caunca et al., 2020; Lim et al., 2020). In the AD group, different from the general quantitative WMHs, CHIPS scores were found to be associated with cingulate lobe atrophy before correction. The cingulate lobe was a part of the cholinergic pathway, which further indicated the cholinergic selectivity of WMHs' impact on brain structures. However, this correlation seemed not meaningful after correction. This might be due to the frequent correlation analysis and the overly conservative correction method. Meanwhile, we used semi-quantitative visual scores to assess the WMHs of the cholinergic pathway, which was less sensitive and less accurate than quantitative measures. In the next study, we will also explore how to quantitatively measure the WMHs of the cholinergic pathway and better explore the association between WMHs and brain atrophy.

Our study suggested that the pathological changes and vascular changes of AD could coexist, and small vascular lesions could probably promote the occurrence of brain atrophy in patients with AD. Compared with previous studies, the strength of this study was that we used a direct measure of the brain atrophy using the brain atrophy ratio (the ratio of the volumes of CSF to brain parenchyma within the respective lobe). This ratio was uniquely provided by AccuBrain^®^ and is not used in other studies. Compared with direct measurement of regional gray matter volume, this ratio could better reduce the diversities caused by distinct individual gray matter volumes and ICV, so as to better reflect the difference in atrophy. We also made automatic segments and measurements of WMHs (including PVWMHs and DWMHs) and corrected WMHs using ICV, thus reducing the impact of different head sizes, which was named WMH ratio in this study. To test the reliability of our WMHr, we also carried out the internationally accepted Fazekas visual rating scale and found that PVWMHr and DWMHr implemented by AccuBrain^®^ were consistent with that rated by Fazekas scales (Supplementary Table 1). Compared with conventional visual rating, this image analysis method was more objective and accurate and could better distinguish the individual difference. In our study, the Spearman's rank correlation coefficients between WMHs and brain atrophy were mostly small, and some of them became meaningless after statistical correction, which meant most of them showed weak correlation or correlation tendency. This might be due to the following: first, the sample size of this study was small so that the conclusion it could respond to was relatively limited; second, the fact that the degree of WMHs in most of the cases we included was light, and the individual differences were small, thus showing a weak correlation in the correlation analysis; third, severe WMHs had been excluded from the common diagnostic criteria of AD, but these individuals might have regional brain atrophy, which made a considerable number of potentially associated individuals missing from our study. Although there was a weak correlation between brain atrophy and WMHs in patients with AD, we could still draw a conclusion that there was a certain degree of connection between WMHs and medial temporal lobe, temporal lobe, cingulate lobe, and insular atrophy. In addition, the average value of MMSE in the AD group was 22.93, which indicated that most patients with AD included in this study were diagnosed with mild AD, which provided a theoretical basis for the early cerebrovascular treatment of AD.

Our research had some limitations. We conducted a cross-sectional study so that we could not confirm whether WMHs had a causal contribution to brain atrophy with age. To further clarify this aspect, prospective longitudinal cohorts should be carried out. In this study, 78.75% of patients with AD were classified as mild cognitive impairment (MMSE ≥ 21); therefore, our conclusion needs to be further verified in moderate to severe AD cases. The AD diagnosis of the samples in our study was not confirmed by pathological biomarkers, so it might not indicate the relationship between WMHs and pathology of Aβ and tau. Due to the heterogeneity of pathological changes in WMHs, to further study the role of WMHs in AD, it is necessary to test the regional WMHs, especially WMHs in the cholinergic pathway, or combine them with pathophysiological characteristics. Clarifying the relationship between WMH and pathological changes of AD is clinically crucial as it may support a new therapy or preventing strategy for AD by controlling cerebrovascular factors.

## Conclusion

White matter hyperintensities are associated with regional brain atrophy in patients with AD, especially with medial temporal lobe, temporal lobe, and insular lobe atrophy. PVWMHs were mainly devoted to these correlations. These cross-sectional findings need to be confirmed by longitudinal studies.

## Data Availability Statement

The original contributions presented in the study are included in the article/Supplementary Material, further inquiries can be directed to the corresponding author/s.

## Ethics Statement

The studies involving human participants were reviewed and approved by Ludwig-Maximilians Universität München Institutional Review Board. The patients/participants provided their written informed consent to participate in this study. Written informed consent was obtained from the individual(s) for the publication of any potentially identifiable images or data included in this article.

## The Alzheimer's Disease Neuroimaging Initiative

Samples included in this study were obtained from the Alzheimer's Disease Neuroimaging Initiative (ADNI) database (adni.loni.usc.edu). A complete listing of ADNI investigators can be found at: http://adni.loni.usc.edu/wp-content/uploads/how_to_apply/ADNI_Acknowledgement_List.pdf. The investigators within the ADNI contributed to the design and implementation of ADNI and provided data but did not participate in analysis or writing of this article.

## Author Contributions

ZC designed the research, organized the data, did the statistical analysis, and prepared and revised the manuscript. YM and WF sorted the data, prepared the figures and tables, and drafted and revised the manuscript. ML, YL, LZ, WL, QY, JX, and YR did the statistical analysis and revised the manuscript. SX, VM, LS, and JL interpreted the results and revised the manuscript. Data used in this study were obtained from the ADNI database but the investigators within the ADNI were uninvolved in the data analysis or drafting of this manuscript. All authors contributed to the article and approved the submitted version.

## Funding

This study was supported by the National Nature Science Foundation of China (Grant Nos. 81870836, 81872261), the Youth Program of National Natural Science Foundation of China (Grant No. 81801083), the China Postdoctoral Science Foundation (2019TQ0384, 2019M660225), the Natural Science Foundation of Guangdong Province (Grant Nos. 2020A1515010210, 2017A030313459), and the Science and Technology Program of Guangzhou, China (Grant No. 202007030010).

## Conflict of Interest

LS is the director of BrainNow Medical Technology Limited. YL and LZ are now employed by BrainNow Medical Technology Limited. The remaining authors declare that the research was conducted in the absence of any commercial or financial relationships that could be construed as a potential conflict of interest.

## Publisher's Note

All claims expressed in this article are solely those of the authors and do not necessarily represent those of their affiliated organizations, or those of the publisher, the editors and the reviewers. Any product that may be evaluated in this article, or claim that may be made by its manufacturer, is not guaranteed or endorsed by the publisher.
