# Supplementary material for: The Correlation Between White Matter Hyperintensity Burden and Regional Brain Volumetry in Patients With Alzheimer's Disease
Source: Front Hum Neurosci. 2022 Jun 14;16:760360. doi: 10.3389/fnhum.2022.760360 (PMC9237397; doi:10.3389/fnhum.2022.760360)
Supplement: Supplementary file 1 [file Table_1.docx]

Supplementary table 1. Correlation analysis of Fazekas score and WMHr

|  | WMHr | | | |
| --- | --- | --- | --- | --- |
|  | PVWMHr | | DWMHr | |
| Fazekas score | *r_S_* | *P* value | *r_S_* | *P* value |
| PVWMHs | 0.526 | **<0.001** | - | **-** |
| DWMHs | - | - | 0.474 | **<0.001** |

Abbreviations: WMHr, quantitative WMHs ratio; WMHs: white matter hyperintensities; PVWMHs, periventricular WMHs; DWMHs, deep WMHs; *r_s_*, Spearman *r*; The *P* value was obtained by Spearman correlation coefficient values. *P*<0.05 was shown in bold.
