# Supplementary material for: The Correlation Between White Matter Hyperintensity Burden and Regional Brain Volumetry in Patients With Alzheimer's Disease
Source: Front Hum Neurosci. 2022 Jun 14;16:760360. doi: 10.3389/fnhum.2022.760360 (PMC9237397; doi:10.3389/fnhum.2022.760360)
Supplement: Supplementary file 2 [file Table_2.docx]

Supplementary table 2. Statistical description after logarithmic transformation

|  |  | NC | AD |  |
| --- | --- | --- | --- | --- |
| **Variables** |  | (n=160) | (n=80) | *P* value |
| DWMHr | Mean(SD), log_10_ ICV% | -1.35(0.52) | -1.26(0.49) | 0.228 |
| PVWMHr | Mean(SD), log_10_ ICV% | -0.85(0.61) | -0.42(0.45) | **<0.001** |
| WMHr | Mean(SD), log_10_ ICV% | -0.69(0.54) | -0.33(0.40) | **<0.001** |

Abbreviations: WMHs, white matter hyperintensities; DWMHs, deep WMHs; PVWMHs, periventricular WMHs; CHIPS, the Cholinergic Pathways Hyperintensities Scale; DWMHr, quantitative DWMHs ratio; PVWMHr, quantitative PVWMHs ratio; WMHr, quantitative WMHs ratio; *P* value<0.05 was shown in bold.
