# Supplementary material for: The Correlation Between White Matter Hyperintensity Burden and Regional Brain Volumetry in Patients With Alzheimer's Disease
Source: Front Hum Neurosci. 2022 Jun 14;16:760360. doi: 10.3389/fnhum.2022.760360 (PMC9237397; doi:10.3389/fnhum.2022.760360)
Supplement: Supplementary file 3 [file Image_1.pdf]

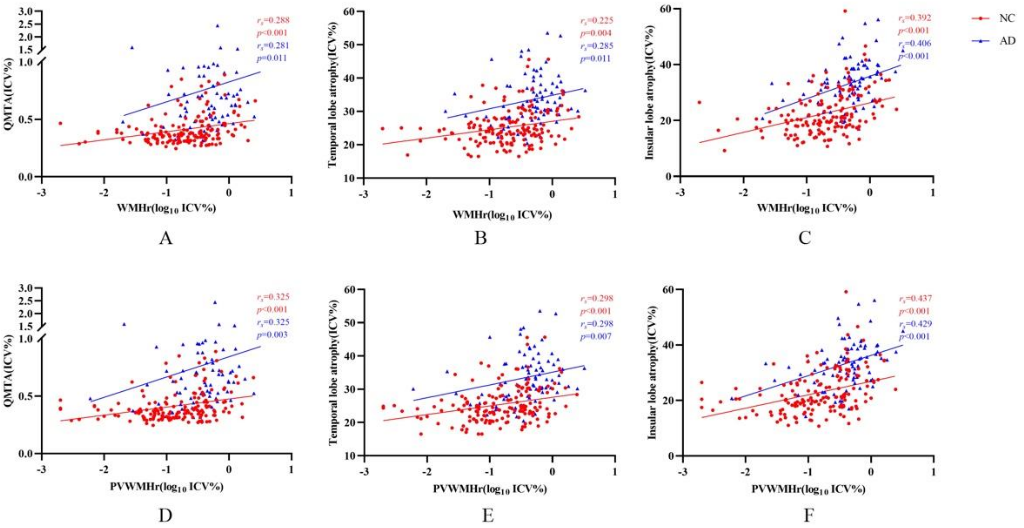

Supplementary figure 1. Scatterplots showing WMHr (A-C) and PVWMHr (D-F) had positive correlation with QMTA, temporal lobe atrophy and insular lobe atrophy in both NC and AD group before Bonferroni correction. r_s and P values obtained by Spearman correlation analysis were also showed in these plots.
